# Genome-Wide Identification of Petunia *HSF* Genes and Potential Function of *PhHSF19* in Benzenoid/Phenylpropanoid Biosynthesis

**DOI:** 10.3390/ijms23062974

**Published:** 2022-03-10

**Authors:** Jianxin Fu, Shuying Huang, Jieyu Qian, Hongsheng Qing, Ziyun Wan, Hefeng Cheng, Chao Zhang

**Affiliations:** Zhejiang Provincial Key Laboratory of Germplasm Innovation and Utilization for Garden Plants, School of Landscape Architecture, Zhejiang Agriculture and Forestry University, Hangzhou 311300, China; 2019105052021@stu.zafu.edu.cn (S.H.); 2019105052025@stu.zafu.edu.cn (J.Q.); qinghongsheng98@163.com (H.Q.); 2020105012012@stu.zafu.edu.cn (Z.W.); chenghf@stu.zafu.edu.cn (H.C.)

**Keywords:** petunia, flower scent, heat shock factor, phenylalanine ammonia-lyase, benzenoid, phenylpropanoid

## Abstract

Volatile benzenoids/phenylpropanoids are the main flower scent compounds in petunia (*Petunia hybrida*). Heat shock factors (HSFs), well known as the main regulator of heat stress response, have been found to be involved in the biosynthesis of benzenoid/phenylpropanoid and other secondary metabolites. In order to figure out the potential function of HSFs in the regulation of floral scent in petunia, we systematically identified the genome-wide petunia *HSF* genes and analyzed their expression and then the interaction between the key petunia *HSF* gene with target gene involved in benzenoid/phenylpropanoid biosynthesis. The results revealed that 34 *HSF* gene family members were obtained in petunia, and most petunia *HSFs* contained one intron. The phylogenetic analysis showed that 23 petunia HSFs were grouped into the largest subfamily HSFA, while only two petunia HSFs were in HSFC subfamily. The DBD domain and NLS motif were well conserved in most petunia HSFs. Most petunia *HSF* genes’ promoters contained STRE motifs, the highest number of *cis*-acting element. *PhHSF19* is highly expressed in petal tubes, followed by peduncles and petal limbs. During flower development, the expression level of *PhHSF19* was dramatically higher at earlier flower opening stages than that at the bud stage, suggesting that *PhHSF19* may have potential roles in regulating benzenoid/phenylpropanoid biosynthesis. The expression pattern of *PhHSF19* is positively related with *PhPAL2*, which catalyzes the first committed step in the phenylpropanoid pathway. In addition, there are three STRE elements in the promoter of *PhPAL2*. *PhHSF19* was proven to positively regulate the expression of *PhPAL2* according to the yeast one hybrid and dual luciferase assays. These results lay a theoretical foundation for further studies of the regulation of HSFs on plant flower scent biosynthesis.

## 1. Introduction

Heat shock factors (HSFs) are well known for their functions as the main regulator of heat stress response [1]. Plant HSFs are encoded by a multi-gene family, and their protein structure is highly conserved and generally contains five core domains: DNA binding domain (DBD), oligomerization domain (OD), nuclear localization signal (NLS), nuclear export signal (NES), and C-terminal short activator peptide motif (AHA motif) [2]. Three evolutionary-conserved classes of HSFs (classes A, B, and C) are divided primarily based on the structural features of OD [3]. DBD is located close to the N-terminus of all HSFs, and consists of three α-helices and four anti-parallel β-sheets. The hydrophobic core of DBD is a helix-turn-helix motif (H2-T-H3), the functional region for transcriptional activation to recognize and bind highly conserved heat stress elements (HSE: 5′-nGAAnnTTCn-3′) [3,4]. Though binding to the HSE motif, plant HSFs can regulate the transcription of potential downstream genes, including, but not limited to, classical heat shock protein genes (*HSPs*) [5,6,7]. In addition, *Arabidopsis* HsfA1a also has a high affinity to stress responsive element (STRE: 5′-AGGGG-3′) and modified HSE motifs (gap-type and TTC-rich-type) in the promoters of target genes [5,7].

Plant HSFs play essential roles in both heat-stress defenses and other abiotic stress tolerance in an HSPs-dependent manner [8,9]. HSFs have also shown their versatile roles in biotic stress, such as powdery mildew infection [10]. Besides their roles in stress responses, HSFs are also involved in plant growth and development [11,12]. Plenty of research has revealed that even under normal growth conditions, plant HSFs participate in organ development, secondary mechanisms, and other life activities. For example, MtHSFA9 from *Medicago truncatula* regulates the depth of dormancy seeds and the biosynthesis of flavonoid, carotenoid, and other secondary metabolites [13]. HSFs have also been found to perform an important function in flower development in lettuce (*Lactuca sativa*), Asteraceae [6]; moso bamboo (*Phyllostachys edulis*), Poaceae [11]; carnation (*Dianthus caryophyllus*), Caryophyllaceae [12]; and wintersweet (*Chimonanthus praecox*), Calycanthaceae [14]. What is more, in *Narcissus tazetta*, 10 members from *HSF* transcription factor family are highly expressed in corona at full-opening stage, when the emission of floral scent (mainly including phenethyl acetate and benzyl acetate) reaches a maximum. The transcriptome-based WGCNA analysis indicates that those *HSFs* are possibly involved in the regulation of biosynthesis and the emission of benzenoid/phenylpropanoid metabolites in *Narcissus tazetta* flowers [15]. Up until now, the regulation of *HSFs* regarding floral scent in other species has not been clear.

Petunia (*Petunia hybrida*) is a kind of ornamental plant widely used as a garden and potted flower. Some petunia cultivars, such as “Mitchell”, emit a strong fragrance at night [16], which is dominated by benzenoid/phenylpropanoid compounds derived from the amino acid phenylalanine [17,18]. As high-quality whole genome sequences of petunia wild parents (*P. axillaris* and *P. inflata*) have been published [19], it was easily found that HSF-binding elements HSE and/or STRE exist in the promoters of most benzenoid biosynthetic genes from their genomes (SNG, https://www.solgenomics.net/ (accessed on September 2021)), such as *PAL* (encoding phenylalanine ammonia-lyase), *BSMT* (encoding benzoic acid/salicylic acid carboxyl methyltransferase), *PAAS* (encoding phenylacetaldehyde synthase), *BPBT* (encoding benzoyl-CoA:benzyl alcohol/2-phenylethanol benzoyltransferase), and *IGS1* (encoding isoeugenol synthase), which suggests that HSFs are possibly capable of manipulating the transcription of those benzenoid biosynthetic genes to affect floral bouquet emissions in petunia.

PAL catalyzes the first committed step in the phenylpropanoid pathway. Considering the contribution of PAL to the regulation of the carbon flux from the amino acid phenylalanine to downstream products of the secondary metabolism, *PAL* genes were predicted to be the key targets of many transcription factors, such as ODORANT1 (ODO1), EMISSION OF BENZENOIDS I (EOBI), and EOBII, which are positive regulators of benzenoid/phenylpropanoid biosynthesis and emissions [16,20,21]. *PAL1* (GenBank accession number AY705976) and *PAL2* (CO805160), two gene members of petunia PALs, have been proven to be target genes of positive transcription factors ODORANT1 (ODO1) and EMISSION OF BENZENOIDS I (EOBI) [16,21]. Additionally, *PAL2* can be regulated by EOBII, but *PAL1* cannot [20].

The present study was carried out to prove the hypothesis of whether *HSF* genes have a potential function in the regulation of benzenoid/phenylpropanoid biosynthesis in petunia. First, we systematically identified and characterized the petunia *HSF* genes. Based on transcriptomic data and qRT-PCR analysis, *PhHSF19* was found as a potential effector involved in petunia benzenoid/phenylpropanoid biosynthesis. Furthermore, *PhHSF19* was proven to positively regulate the expression of *PhPAL2*. Our results will provide valuable additional information for the functional analysis of *HSF* genes in petunia.

## 2. Results

### 2.1. Identification of HSF Gene Family Members in Petunia

Through domain verification, 34 *HSF* gene family members were obtained in petunia. The length of the 34 HSF proteins varied from 69 (Peaxi162Scf00205g00820) to 512 (Peaxi162Scf00045g00131) amino acids, while the molecular weight ranged from 8.02 kD to 56.56 kD (Table 1). The theoretical isoelectric point was between 4.32 to 9.42. Furthermore, except one HSF protein (Peaxi162Scf00205g00820), the total average hydrophobic index of other HSF proteins was negative, suggesting that most HSF proteins belonged to hydrophilic proteins. The aliphatic index is the relative content of aliphatic side chains, including alanine, valine, isoleucine, and leucine. The aliphatic index of HSF proteins was from 54.71 (Peaxi162Scf00450g00422) to 94.49 (Peaxi162Scf00205g00820). All HSFs were predicted to be located in the nucleus.

### 2.2. Gene Structure, Phylogeny Analysis, and Conserved Motifs of Petunia HSFs

**Figure 1:**
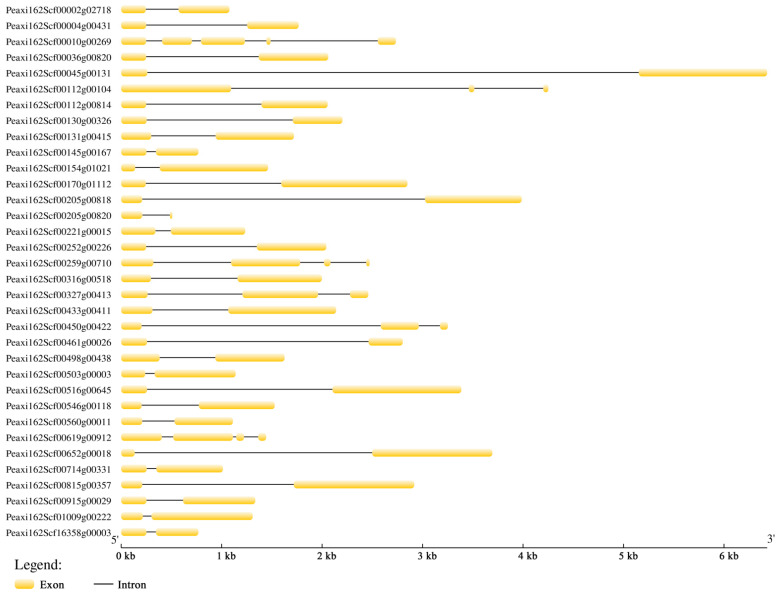
Gene structure analysis of 34 petunia HSFs. The exons and introns are indicated by the yellow boxes and black lines, respectively.

The intron and exon structures were analyzed using GSDS 2.0 to reveal the gene structural features of petunia *HSFs* (Figure 1). Out of 34 petunia *HSFs*, 28 genes contained two exons and one intron. The other six genes had three to five exons. Peaxi162Scf00045g00131 had the longest sequence (6430 bp), while Peaxi162Scf00205g00820 had the shortest (477 bp).

**Figure 2:**
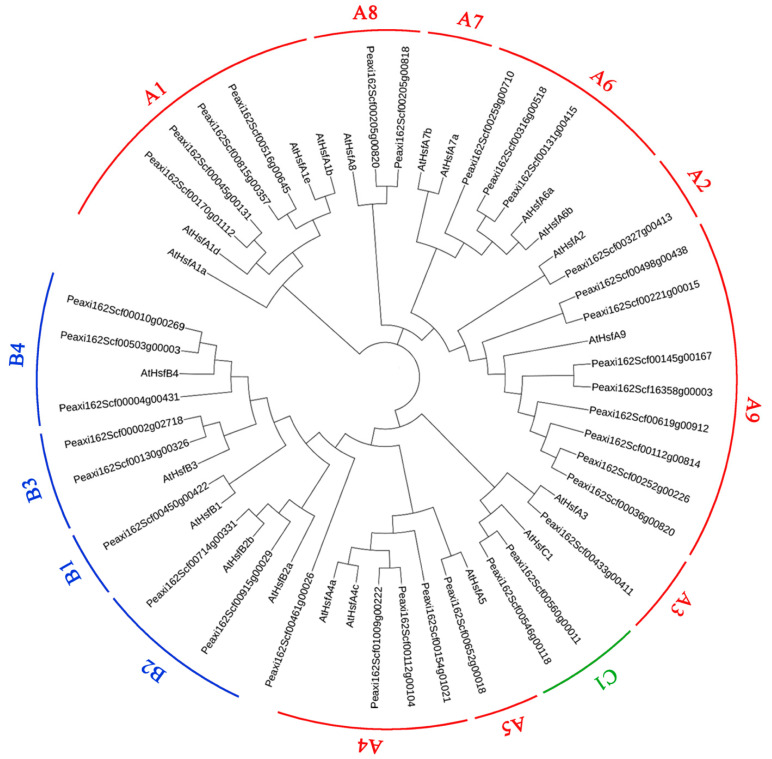
Phylogeny analysis of petunia HSFs with AtHSFs from *Arabidopsis thaliana*. HSFs were divided into three major subfamilies HSFA (red), HSFB (blue), and HSFC (green).

Based on well-established Arabidopsis HSF family classification [22], petunia HSFs were also divided into three major subfamilies with 21 HSF proteins from *A. thaliana*, HSFA (red), HSFB (blue), and HSFC (green), which is shown in Figure 2. The HSFA subfamily is the largest with 23 petunia HSFs, while there are only two petunia HSFs in the HSFC subfamily. Among the HSFA subfamily, eight HSFs belong to class A9, followed by four HSFs in classes A1. However, none of the petunia HSFs belongs to class A7.

**Figure 3:**
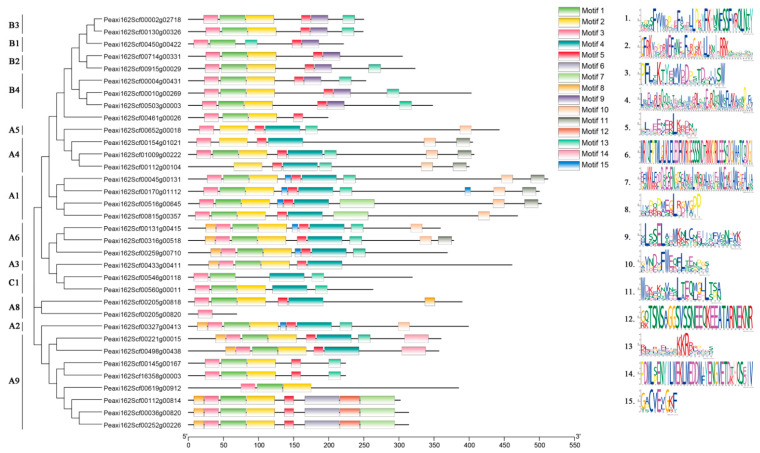
Conserved motif analysis of petunia HSFs. Proteins are organized according to the groups in Figure 2. Fifteen motifs are found in the protein sequences.

Fifteen conserved motifs in petunia HSFs were identified and analyzed using the online software MEME (Figure 3). Motif 1, motif 2, and motif 3 in the N-terminals contained 37, 41, and 29 amino acids, respectively. Motifs 1–3 constituted the DBD domain (~100aa) and were widely distributed and highly conserved in most petunia HSFs. In addition, 20 out of 34 HSF proteins had motif 13, referring to the NLS motif. In addition, motif 4 was observed in the HSFs from subfamily A and subfamily C, but was not included in subfamily B. Motif 9 was only present in subfamily B. Motif 5 was found in HSFs from subfamily A and subfamily B, rather than subfamily C. AHA motif (motif 10) and NES motif (motif 11) only existed in subfamily A.

### 2.3. Cis-Acting Elements Analysis of Petunia HSFs

**Figure 4:**
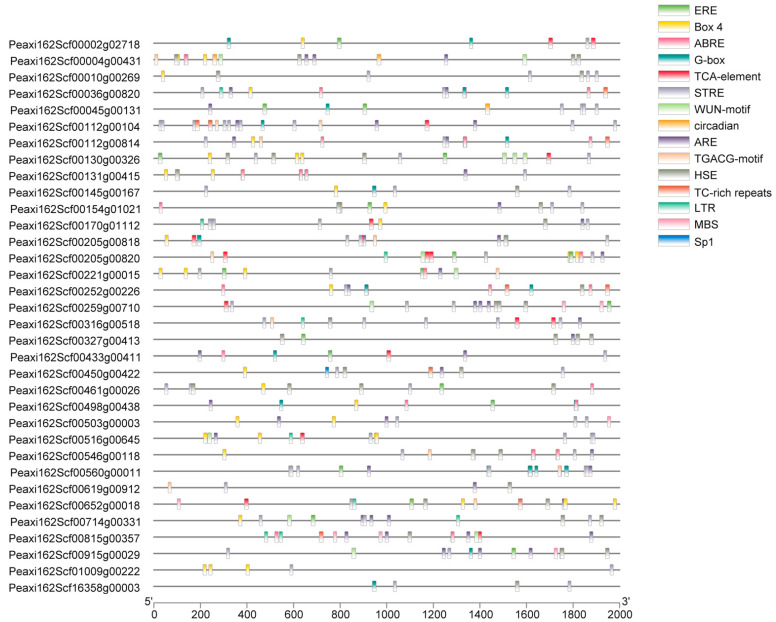
*Cis*-acting elements analysis of promoter sequences of petunia HSFs. The position of *cis*-acting elements in the 2kb upstream region is represented by different colored bars.

The PlantCARE online tool was used to analyze the promoter *cis*-acting elements of 34 petunia HSFs, and the core *cis*-acting elements in each promoter are shown in Figure 4. It was predicted that promoters of petunia *HSFs* contained a variety of hormone-response *cis*-acting elements, such as ABA-response element ABRE, methyl jasmonate response element TGACG, salicylic acid response element TCA, and ethylene response element ERE. Several well-known stress-related elements, including HSE, STRE, TC-rich, LTR-motif, ARE, and MBS, were found in the promoters of 34 petunia HSFs. In addition, promoters of petunia *HSFs* also possessed light stress response elements (Box 4, G-box, and Spl), wounding responsive element WUN, and circadian element. Among these mentioned elements, 28 out of 34 petunia *HSF* promoters contained STRE motifs, the highest number of *cis*-acting elements. There were six STRE motifs in the promoter of Peaxi162Scf00560g00011, followed by Peaxi162Scf00170g01112 and Peaxi162Scf00316g00518 with five STRE motifs. Except for the STRE motif, the other three *cis*-acting elements, ARE, Box 4, and HSE, had a higher frequency in the promoters of 34 *HSFs*. There were four HSE motifs in the promoters of Peaxi162Scf00004g00431, Peaxi162Scf00327g00413, and Peaxi162Scf00461g00026.

### 2.4. Selection of Potential Key Petunia HSF Genes Based on RNA Sequencing and qRT-PCR Analysis

**Figure 5:**
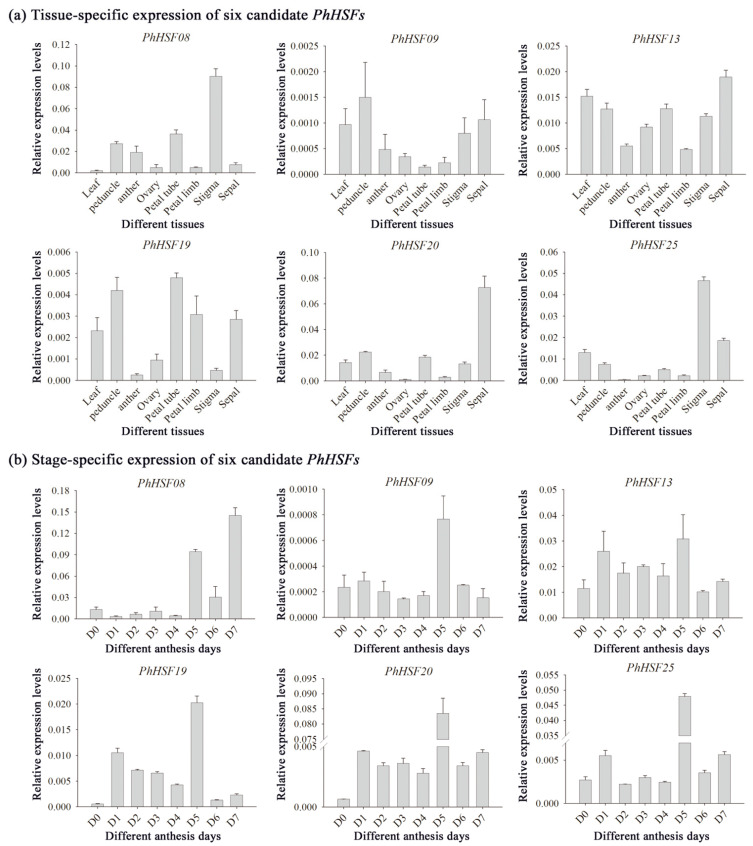
qRT-PCR analysis of six candidate *PhHSF* genes. All transcript levels were determined by qRT-PCR and were obtained relative to the reference gene (*PhEF1-α*). (**a**) Tissue-specific expression of *PhHSFs* in leaf, peduncle, anther, ovary, petal tube, petal limb, stigma, and sepal collected at 1500 h of day 1 post-anthesis (D1). (**b**) Stage-specific expression of *PhHSFs* from mature buds (D0) to day 7 post-anthesis (D7) at 1500 h. Representative histograms from multiple biological replications using the 2^−∆CT^ method are shown (mean ± SE, *n* = 3).

Based on the reported RNA sequencing data of the day 0 (bud) or day 2 (post-anthesis) corolla tissue of *P. hybrida* “Mitchell” [23], 33 assembled sequences could be mapped to the *HSF* genes of *Petunia axillaris*, and were named *PhHSF01*-*PhHSF33* (Table S1). Among them, six *PhHSFs* with a fold-change of day 2 vs. day 0 ≥ 2 and the expression level (RPKM) at day 2 ≥ 1000, including *PhHSF08*, *PhHSF09*, *PhHSF13*, *PhHSF19*, *PhHSF20*, and *PhHSF25*, were predicted to have potential roles in regulating benzenoid/phenylpropanoid biosynthesis (Table S1). The expression levels of six potential key *PhHSF* genes in different tissues and during the flower opening period were detected by qRT-PCR. The results showed that *PhHSF19* is highly expressed in petal tubes, followed by stems and petal limbs (Figure 5a). However, the expression level of the other five *PhHSF* genes was much lower in the petal limbs—the main tissues emitting volatile benzenoid/phenylpropanoid in petunia [24]. During flower development, the expression level of *PhHSF19* in D1 increased 19.4-fold compared to that in D0 (Figure 5b). It gradually decreased from D1 to D4, but dramatically increased in D5, and then sharply decreased. Taking the expression patterns of *PhHSF19* in different tissues and at different flower development into consideration, *PhHSF19* probably had a positive effect on volatile benzenoid/phenylpropanoid biosynthesis and was chosen for further biological function analysis.

### 2.5. Y1H and Dual Luciferase Assays

**Figure 6:**
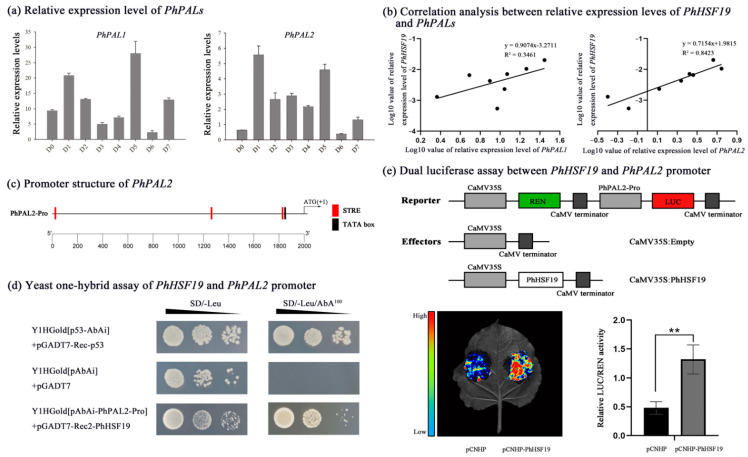
*PhHSF19* could bind to the *PhPAL2* promoter. (**a**) Relative expression level of *PhPAL1* and *PhPAL2* during flower opening. (**b**) Correlation analysis between the relative expression of *PhHSF19* and *PhPAL1*/*PhPAL2*. (**c**) Location of the STRE *cis*-element in the *PhPAL2* promoter. (**d**) Yeast one-hybrid assays of the *PhHSF19* and *PhPAL2* promoters. (**e**) Dual luciferase assay between the *PhHSF19* and *PhPAL2* promoter, including the schematic diagram of the double-reporter and effector plasmids, qualitative results, and quantitative results. T-test was used for statistical analyses of quantitative results compared with corresponding control (**, *p* < 0.01).

When all kinds of benzenoids/phenylpropanoids accumulated to high levels during early flower opening period [25], the expression level of *PhPAL2* dramatically increased, while that of *PhPAL1* changed slightly (Figure 6a), revealing that the *PhPAL2* gene would play a more important role in volatile benzenoid/phenylpropanoid biosynthesis, compared with *PhPAL1*. The expression pattern of *PhHSF19* was positively related with *PhPAL2* (R^2^ = 0.8423), rather than *PhPAL1* (Figure 6b). There were three STRE elements in the promoter of *PhPAL2* located at nucleotides −1951, −750, and −172 from the initial ATG codon (Figure 6c). We first performed the Y1H assay to address whether PhHSF19 could regulate the expression of *PhPAL2*. Only co-transformed yeasts of pGADT7-Rec2-PhHSF19 and pAbAi-PhPAL2-Pro could survive and grow normally on the selective medium of SD/-Leu/100 ng mL^−1^ AbA, but the control did not survive (Figure 6d), revealing that PhHSF19 can interact with *PhPAL2* promoter in the yeast system. In addition, a dual-luciferase assay was employed to further verify the regulation of PhHSF19 on the *PhPAL2* promoter. As shown in Figure 6e, a stronger fluorescent signal was detected in the tobacco leaf co-transformed with pCNHP-PhHSF19 plus pGreenII-PhPAL2-Pro-LUC compared to the control, and the LUC/REN ratio of tobacco co-transformed with the effector containing PhHSF19 and the reporter containing promoter of *PhPAL2* were drastically enhanced with an approximate 2.8-fold induction.

## 3. Discussion

HSFs act as essential regulators in various abiotic stresses [1]. There are 21 *HSF* gene members in *Arabidopsis* [22], 25 members in rice [22], 31 members in *Populus trichocarpa* [26], and 17 members in *Dianthus caryophyllus* [12]. In this study, a total of 34 *HSFs* were identified in the petunia genome, which is greater than the number of *HSF* gene members in the four above-mentioned plant species. Similar to other species, most petunia HSFs belong to the HSFA subfamily. There are eight petunia *HSF* members in class A9, while only one or two members of the same class are found in *Arabidopsis*, *P. trichocarpa*, and *D. caryophyllus* [12,22,26]. Additionally, class A7 is absent in petunia. These results suggest that gene loss and gene duplication of *HSFs* occur in the evolution process of petunia.

Besides motifs 1–3 (DBD domain) and motif 13 (NLS motif), which are in almost all petunia HSFs, some conserved motifs are present in specific HSF subfamilies. AHA motif (motif 10), which functions as a transcription activator [1], only exists in subfamily A, suggesting that *HSF* members from subfamily B and subfamily C might probably lack transcriptional activation function. Similar to the AHA motif, the NES motif (motif 11) is also only found in the HSFs from subfamily A. OD, called the HR-A/B region, can be used to distinguish subfamily B from subfamilies A and C [1], as this domain in petunia is longer (as motif 4) in subfamilies A and C, but is less (as motif 9) in subfamily B, which is more similar to other species [27,28,29].

In addition to their roles in stress responses [30,31], HSFs are also involved in plant development [6,32] and secondary mechanism [5,15]. Several *HSFs* in *Narcissus tazetta* are predicted to have an effect on flower scent emission [15]. HSF-binding elements HSE and/or STRE are present in the promoters of many benzenoid biosynthetic genes, which advances the hypothesis that HSFs probably play a vital part in the regulation of benzenoid/phenylpropanoid biosynthesis and emissions in petunia. A previous study revealed that flowers at one day or two days post-anthesis emitted the greatest amount of 12 benzenoids/phenylpropanoids, such as methyl benzoate, benzaldehyde, benzyl benzoate, phenylacetaldehyde, and isoeugenol [25]. *PhHSF19*, with an up-regulated expression during this period based on RNA sequencing and qRT-PCR analysis (Table S1 and Figure 5), was identified as a potential key *HSF* gene in benzenoid/phenylpropanoid biosynthesis for further research.

PAL is involved in the production of precursors which contribute to benzenoid/phenylpropanoid biosynthesis [17]. Three STRE elements, the important *cis*-acting regulatory elements that HSF can bind to, are present in the promoter of the *PhPAL2* gene (Figure 6c). What is more, *PhPAL2* was chosen as a candidate target gene of PhHSF19 because of their similar expression pattern. The expression level of *PhPAL2* is significantly higher at the early flower opening stage than that at the bud stage (Figure 6a), and the expression pattern of *PhHSF19* is positively related with *PhPAL2*, rather than *PhPAL1* (Figure 6b). Both *PhPAL1* and *PhPAL2* from the *PAL* gene family are supposed to have a similar and redundant function, but the expression pattern of these two genes is not the same, suggesting that *PhPAL1* and *PhPAL2* exhibit a different response to the regulation of different transcription factors. In this study, *PhPAL2* was the candidate target gene of *PhHSF19*, while *PhPAL1* was not. Similarly, *PhPAL2* can be regulated by EOBII, but *PhPAL1* cannot [20].

The phylogeny analysis showed that *PhHSF19* belonging to class A3 without AHA motifs does not perform as a transcription activator [1]. However, a previous study found that tryptophane residues in the C-terminal domains of HSFs from class A3 provide an additive contribution to the activator function [33]. The important tryptophane residues are also present in the C-terminal domains of PhHSF19, and we speculated that PhHSF19 should possess a transcriptional activation function. Then, the Y1H and dual luciferase assays not only prove this speculation, but also confirmed the interaction between *PhHSF19* and *PhPAL2* in our study. Based on the dual luciferase assay, about a 2.8-fold induction in the LUC/REN ratio verified that *PhHSF19* could positively regulate the expression of *PhPAL2*. Therefore, an up-regulated expression of *PhPAL2* under the regulation of *PhHSF19* would enhance the transformation of phenylalanine to *trans*-cinnamic acid [17], which probably provided more metabolic substrates for further benzenoid biosynthesis and made the flower fragrance more intense. What is more, LsHSFB2A-1 can bind to the LsMADS55 promoter to regulate floral organ specification and flower development in lettuce [6]. The expression levels of *DcaHsf-A5* and *DcaHsf-B2b* are closely related with flower development and are the highest at the full-opening stage (FS6) [12]. This suggests that PhHSF19 might have a similar function in the flower opening process, which would also affect flower scent to a certain extent. More research needs be carried out to prove this assumption.

In summary, 34 *HSF* gene family members were identified in petunia. The expression analysis suggested that *PhHSF19* possesses potential roles in regulating benzenoid/phenylpropanoid biosynthesis. In addition, PhHSF19 could positively regulate the expression of *PhPAL2* based on the yeast one hybrid and dual luciferase assays.

## 4. Materials and Methods

### 4.1. Plant Materials

*Petunia hybrida* “Mitchell” plants were cultured in an environmental chamber in Zhejiang Agriculture and Forestry University, Hangzhou, China. The chamber was programmed for 16 h light/8 h dark with a temperature of 24 °C, with the light period starting at 07:00 a.m. Petal limbs were sampled at 16:00 p.m. from three individual flowers before anthesis (day 0, D0) and from the first to seventh day of anthesis (D1–D7). In addition, different tissues, including leaf, peduncle, anther, ovary, petal tube, petal limb, stigma, and sepal of three individual plants at 16:00 p.m. were collected on the second day of anthesis. All of the samples were immediately frozen in liquid nitrogen and stored at −80 °C.

### 4.2. Identification HSF Genes in Petunia

The Hidden Markov Model (HMM) of the HSF-type DBD domain (PF00447) was obtained from the Pram database (http://pfam.xfam.org (accessed on September 2021)) and was used to identify the petunia *HSF* genes. HMMER was involved as it was extracted from the candidate gene of the *HSF* gene family from *Petunia axillaris* genome (SNG, https://www.solgenomics.net/ (accessed on September 2021)). In order to verify the accuracy of the obtained *HSF* genes, the protein sequences of the *HSF* genes were further confirmed in NCBI-CDD (https://www.ncbi.nlm.nih.gov/Structure/bwrpsb/bwrpsb.cgi (accessed on September 2021)) and the SMART online website (https://smart.embl-heidelberg.de/ (accessed on September 2021)). The online ExPASy program (http://web.expasy.org/protparam/ (accessed on September 2021)) was used to predict the basic physical and chemical properties, such as the amino acid sequence length, protein molecular weight, and theoretical isoelectric point (pI) of each petunia HSF protein. The subcellular localization of petunia *HSFs* was analyzed using PSORT (https://www.genscript.com/psort.html/ (accessed on September 2021)).

### 4.3. Gene Structure and Phylogenetic Analysis of Petunia HSFs

The exon/intron structures of *PhHSFs* were displayed by the Gene Structure Displayer Server v2.0 (GSDS, http://gsds.cbi.pku.edu.cn/ (accessed on September 2021)). The amino acid sequences of 21 AtHSFs from *Arabidopsis thaliana* were gathered from the TAIR website (http://www.arabidopsis.org/ (accessed on September 2021)). MEGA6.0 was used for the multiple sequence alignment of the amino acid sequences of the Arabidopsis and petunia HSFs. The phylogenetic tree was constructed using the maximum likelihood method with 1000 replicates.

### 4.4. Conserved Motifs and Cis-Acting Elements Analysis of Petunia HSFs

Multiple Expectation maximization for Motif Elicitation (MEME, http://meme-suite.org/ (accessed on September 2021)) was used to analyzed the conserved motifs of each gene protein, with the following the parameters: the minimum length of conservative motifs was 6, the maximum length was 50, the maximum number of conservative motifs found was 15, and other parameters were default values. The 2000-bp sequence in the 5’-flanking upstream region of the initiation codon of each petunia HSF was retrieved by Phytozome (https://phytozome.jgi.doe.gov/jbrowse/index.html (accessed on September 2021)). Then, the PlantCARE database (http://bioinformatics.psb.ugent.be/webtools/plantcare/html/ (accessed on September 2021)) was used to predict the *cis*-acting elements in each promoter sequence.

### 4.5. Selection of Potential Key PhHSF Genes Based on RNA Sequencing

RNA sequencing data for the day 0 (bud) or day 2 (post-anthesis) corolla tissue of *P. hybrida* “Mitchell” were obtained from the NBCI Gene Expression Omnibus (GSE70948) [23]. RNA sequencing analysis was conducted using the same method as in a previous study [34]. The assembled sequences were assigned to *Petunia axillaris* (v1.6.2) genomes [19,35] with Local BLAST to identify *PhHSF* genes, and the corresponding gene expressions were acquired with the reads per kilobases per million reads (RPKM) statistics. Twenty *PhHSF* genes were screened under a threshold of identify of ≥95% (Table S1). Then, six differentially expressed *PhHSF* genes were further determined with a fold-change of day 2 vs. day 0 ≥ 2 and the expression level (RPKM) at day 2 ≥ 1000.

### 4.6. Total RNA Extraction, cDNA Reverse Transcription and qRT-PCR Analysis

The total RNA was extracted from different samples according to the manufacturer’s instruction for the FastPure^®^ Plant Total RNA Isolation Kit (Polysaccharides and Polyphenolics-rich) (Vazyme, Nanjing, China). The first-strand cDNA was synthesized from 500 ng total RNA, 2 μL 5× PrimerScript RT Master Mix, and RNase Free ddH_2_O to 10 μL reaction mixture using the PrimeScript™ RT Master Mix (Takara, Dalian, China). The relative expression of the related genes (six differentially expressed *PhHSF* genes and two *PhPALs*) was determined by qRT-PCR using ChamQ SYBR qPCR Master Mix (Vazyme, Nanjing, China) on LightCycler^®^ 480 II (Roche Diagnostics, Mannheim, Germany). The 10 μL reaction condition was configured according to the following instruction: 95 °C for 30 s, 40 cycles of amplification (95 °C for 10 s, 60 °C for 30 s, and 60 °C for 30 s), and melting curve (95 °C for 5 s, 60 °C for 1 min, and then 95 °C). Primer sequences are shown in Table S2. The relative expression of the target genes was calculated with 2^−∆CT^ using the reference gene *PhEF1-α*.

### 4.7. Y1H Assay

The yeast one hybrid assay was used to identify the transcriptional regulators of *PhHSF19*. The promoter sequence of *PhPAL2* (PhPAL2-Pro) and was amplified and inserted into the pAbAi vector, and the CDS of *PhHSF19* was cloned into the pGADT7-Rec2 vector with the primers listed in Table S2. pAbAi-PhPAL2-Pro was transformed into Y1H Gold according to the protocol of Yeastmaker™ Yeast Transformation System 2 User Manual, and the lowest inhibitory concentration of aureobasidin A (AbA) for the bait strain was tested on SD/-Ura media. The prey was transformed into yeast cells containing recombinant pAbAi vector and then incubated on SD/-Leu medium at 30 °C for 3 days. The interactions between pGADT7-Rec2-PhHSF19 and pAbAi-PhPAL2-Pro were detected on an SD/-Leu medium with 100 ng mL^−1^ AbA at 30 °C for 3–5 days.

### 4.8. Dual Luciferase Assay

A dual-luciferase assay was used to further test the interaction between *PhHSF19* and the promoter of the *PhPAL2* in vivo. Full-length sequence of regulatory genes and target promoters were cloned into pCNHP and pGreenII 0800-LUC vectors, respectively, using the primers listed in Table S2. Then, the recombinant vectors were transformed into *Agrobacterium* strain GV3101 (pSoup). The transformed strains were cultured in 28 °C and then resuspended in an infiltration medium (10 mM MgCl_2_ and 200 μM acetosyringone) to an OD 600 of 1.6. The cell suspension was mixed at a ratio of 1:1 (*v/v*) and injected into *Nicotiana benthamiana* leaves with needleless syringes. After 3 days, the transformed tobacco leaves, which had been sprayed with D-luciferin sodium salt (Coolaber, Beijing, China), were observed under Fuison-FX7 multi-imaging apparatus (Vilber, Collégien, France).

## Figures and Tables

**Table 1:** Characteristics of petunia heat shock transcription factors, i.e., protein length (aa), molecular weight (MW), theoretical isoelectric point (pI), instability index, hydrophilic index, aliphatic index, and predicted subcellular localization.

Loci	Protein Length (aa)	Molecular Weight (kD)	Theoretical pI	Instability Index	Hydrophilic Index	Aliphatic Index	Subcellular Localization
Peaxi162Scf00002g02718	250	28.83	5.77	55.15	−0.868	64.40	nucleus
Peaxi162Scf00004g00431	253	29.36	6.56	41.27	−0.745	84.31	nucleus
Peaxi162Scf00010g00269	403	45.91	6.34	60.36	−0.734	70.89	nucleus
Peaxi162Scf00036g00820	314	35.82	6.12	47.69	−0.805	72.07	nucleus
Peaxi162Scf00045g00131	512	56.56	4.71	68.62	−0.636	68.57	nucleus
Peaxi162Scf00112g00104	400	45.40	5.48	47.29	−0.649	79.20	nucleus
Peaxi162Scf00112g00814	302	34.25	5.99	42.22	−0.728	75.56	nucleus
Peaxi162Scf00130g00326	249	28.91	6.68	54.38	−0.865	65.30	nucleus
Peaxi162Scf00131g00415	359	41.74	5.00	51.96	−0.835	64.32	nucleus
Peaxi162Scf00145g00167	224	25.98	9.18	41.12	−0.728	74.06	nucleus
Peaxi162Scf00154g01021	405	46.04	5.39	54.92	−0.820	71.23	nucleus
Peaxi162Scf00170g01112	500	55.63	4.75	67.61	−0.647	66.08	nucleus
Peaxi162Scf00205g00818	390	45.15	4.63	54.33	−0.673	73.46	nucleus
Peaxi162Scf00205g00820	69	8.02	4.32	42.50	0.333	94.49	nucleus
Peaxi162Scf00221g00015	360	41.84	6.64	57.17	−0.759	70.92	nucleus
Peaxi162Scf00252g00226	314	35.81	5.35	48.60	−0.803	71.43	nucleus
Peaxi162Scf00259g00710	369	42.10	5.42	51.46	−0.751	65.77	nucleus
Peaxi162Scf00316g00518	378	44.14	5.08	49.36	−0.883	67.57	nucleus
Peaxi162Scf00327g00413	399	45.58	5.19	53.32	−0.406	87.39	nucleus
Peaxi162Scf00433g00411	461	51.58	4.90	54.28	−0.526	66.18	nucleus
Peaxi162Scf00450g00422	221	24.71	5.85	41.11	−0.765	54.71	nucleus
Peaxi162Scf00461g00026	199	23.31	9.42	49.99	−0.735	69.10	nucleus
Peaxi162Scf00498g00438	357	41.59	5.08	55.61	−0.777	70.73	nucleus
Peaxi162Scf00503g00003	348	39.75	7.75	52.24	−0.664	64.91	nucleus
Peaxi162Scf00516g00645	503	55.67	5.67	47.46	−0.604	74.77	nucleus
Peaxi162Scf00546g00118	319	36.05	5.35	69.94	−0.600	63.86	nucleus
Peaxi162Scf00560g00011	263	30.25	6.62	47.95	−0.651	64.52	nucleus
Peaxi162Scf00619g00912	385	45.37	5.43	42.70	−0.907	60.08	nucleus
Peaxi162Scf00652g00018	443	49.37	5.39	57.72	−0.794	65.80	nucleus
Peaxi162Scf00714g00331	305	33.23	5.24	56.40	−0.341	80.89	nucleus
Peaxi162Scf00815g00357	469	52.79	5.40	49.77	−0.550	72.69	nucleus
Peaxi162Scf00915g00029	323	35.67	4.94	53.00	−0.667	68.85	nucleus
Peaxi162Scf01009g00222	407	46.63	5.20	48.51	−0.754	70.42	nucleus
Peaxi162Scf16358g00003	224	25.90	9.19	41.46	−0.729	74.06	nucleus

## Data Availability

Not applicable.

## Supplementary Materials

The following supporting information can be downloaded at: https://www.mdpi.com/article/10.3390/ijms23062974/s1.
