# Supplementary material for: Genome-Wide Identification of Petunia HSF Genes and Potential Function of PhHSF19 in Benzenoid/Phenylpropanoid Biosynthesis
Source: Int J Mol Sci. 2022 Mar 10;23(6):2974. doi: 10.3390/ijms23062974 (PMC8951162; doi:10.3390/ijms23062974)
Supplement: Supplementary file 1 [file ijms-23-02974-s001.zip › ijms-1617692-supplementary/Table S1 Expression of PhHSF genes based on RNA sequencing.pdf]

Table S1 Expression of *PhHSF* genes based on RNA sequencing.Including bud expression level (RPKM)and the average, day2 expression level (RPKM) and the average, change fold, identity and evalue.

Gene name	Loci	Bud_Rep 1	Bud_Rep 2	Bud_Rep 3	Bud_average	Day2_Rep 1	Day2_Rep 2	Day2_Rep 3	Day2_ average	Change fold	Identity %	evalue
PhHSF01	Peaxi162Scf00002g02718	701	547	638	628.67	41	62	66	56.33	0.09	100	0
PhHSF02	Peaxi162Scf00004g00431	33	24	21	26.00	0	0	0	0.00	0.00	99.209	0
PhHSF03	Peaxi162Scf00010g00269	10	19	33	20.67	0	0	2	0.67	0.03	99.695	0
PhHSF04	Peaxi162Scf00036g00820	1380	1147	1519	1348.67	972	1281	1228	1160.33	0.86	40.764	2.95E-31
PhHSF05	Peaxi162Scf00045g00131	766	707	855	776.00	763	954	826	847.67	1.09	98.047	0
PhHSF06	Peaxi162Scf00112g00104	496	407	633	512.00	1278	1230	1143	1217.00	2.38	94.215	0
PhHSF07	Peaxi162Scf00112g00814	1380	1147	1519	1348.67	972	1281	1228	1160.33	0.86	41.401	1.04E-31
PhHSF08	Peaxi162Scf00130g00326	161	100	314	191.67	1901	1586	1714	1733.67	9.05	99.598	0
PhHSF09	Peaxi162Scf00131g00415	289	412	712	471.00	1121	816	1141	1026.00	2.18	100	0
PhHSF010	Peaxi162Scf00145g00167	25	27	11	21.00	15	26	11	17.33	0.83	95.27	5.28E-102
PhHSF11	Peaxi162Scf00154g01021	496	407	633	512.00	1278	1230	1143	1217.00	2.38	31.767	2.71E-63

PhHSF12	Peaxi162Scf00170g01112	388	287	476	383.67	251	229	209	229.67	0.60	100	0
PhHSF13	Peaxi162Scf00205g00818	349	379	455	394.33	994	1104	923	1007.00	2.55	100	0
PhHSF14	Peaxi162Scf00221g00015	4	9	10	7.67	8	8	0	5.33	0.70	80.571	2.96E-90
PhHSF15	Peaxi162Scf00252g00226	1380	1147	1519	1348.67	972	1281	1228	1160.33	0.86	41.83	4.09E-31
PhHSF16	Peaxi162Scf00259g00710	1380	1147	1519	1348.67	972	1281	1228	1160.33	0.86	60.19	9.81E-84
PhHSF17	Peaxi162Scf00316g00518	17	20	9	15.33	3	3	4	3.33	0.22	100	0
PhHSF18	Peaxi162Scf00327g00413	148	253	213	204.67	224	682	287	397.67	1.94	100	1.48E-154
PhHSF19	Peaxi162Scf00433g00411	275	265	297	279.00	2387	2350	1406	2047.67	7.34	100	0
PhHSF20	Peaxi162Scf00450g00422	320	240	415	325.00	11329	11222	11715	11422.00	35.14	97.163	5.26E-95
PhHSF21	Peaxi162Scf00461g00026	2	3	0	1.67	0	0	0	0.00	0.00	98.291	1.02E-81
PhHSF22	Peaxi162Scf00498g00438	4	9	10	7.67	8	8	0	5.33	0.70	97.516	1.10E-112
PhHSF23	Peaxi162Scf00503g00003	10	19	33	20.67	0	0	2	0.67	0.03	67.867	1.27E-156
PhHSF24	Peaxi162Scf00516g00645	1380	1147	1519	1348.67	972	1281	1228	1160.33	0.86	99.801	0

PhHSF25	Peaxi162Scf00546g00118	1621	1237	2672	1843.33	5360	3966	6376	5234.00	2.84	98.762	0
PhHSF26	Peaxi162Scf00560g00011	1621	1237	2672	1843.33	5360	3966	6376	5234.00	2.84	68.908	1.76E-111
PhHSF27	Peaxi162Scf00619g00912	1621	1237	2672	1843.33	5360	3966	6376	5234.00	2.84	36.757	3.68E-29
PhHSF28	Peaxi162Scf00652g00018	465	387	476	431.50	748	940.00	744	810.67	1.88	92.827	0
PhHSF29	Peaxi162Scf00714g00331	35	59	28	40.67	11	27	20	19.33	0.48	97.049	0
PhHSF30	Peaxi162Scf00815g00357	243	207	316	255.33	490	556	503	516.33	2.02	100	0
PhHSF31	Peaxi162Scf00915g00029	497	592	581	556.67	403	597	429	476.33	0.86	99.381	0
PhHSF32	Peaxi162Scf01009g00222	496	407	633	512.00	1278	1230	1143	1217.00	2.38	72.727	0
PhHSF33	Peaxi162Scf16358g00003	25	27	11	21.00	15	26	11	17.33	0.83	94.595	1.22E-101
