# Supplementary material for: Genome-Wide Identification of Petunia HSF Genes and Potential Function of PhHSF19 in Benzenoid/Phenylpropanoid Biosynthesis
Source: Int J Mol Sci. 2022 Mar 10;23(6):2974. doi: 10.3390/ijms23062974 (PMC8951162; doi:10.3390/ijms23062974)
Supplement: Supplementary file 1 [file ijms-23-02974-s001.zip › ijms-1617692-supplementary/Table S2 Primer sequence.pdf]

Table S2 Primer sequence used in Y1H assay and Dual luciferase assay

Primer name	Primer sequence(5'-3')
PGADT7-Rec2-PhHSF19-F	CCATGGAGGCCAGTGAATTCATGAATCCATTTGATAATACTGAACTGAAATGGAC
PGADT7-Rec2-PhHSF19-R	CCACTGCTTGGGTGGCTAAAAACTATCATTCTTGGGCTGGCTG
pCNHP-PhHSF19-F	TGCCCAAATTCGCGCCATGAATCCATTTGATAATACTGAAACTGAAATGGAC
pCNHP-PhHSF19-R	AAAATTTAATGAAACCAGAGTTAACCTAAAAACTATCATTCTTGGGCTGGCTG
pAbAi-PhPAL2 PRO-F	GTACCCGGGGATCTGTCGACTATTCCCCGATGGCCTTGATTCGTGAG
pAbAi-PhPAL2 PRO-R	GCACATGCCTCGAGGTGGATCAAGAAAATGTTTATTAAGGGTGGTAGAAATG
pGreenII0800-PhPAL2 PRO-F	GGTCGACGGTATCGATATATTCCCCGATGGCCTTGATTCG
pGreenII0800-PhPAL2 PRO-R	CCGCTCTAGAACTAGTGTGGATCAAGAAAATGTTTATTAAGGGTGGTAGAAATG
